# Physiological Effects of Nature Therapy: A Review of the Research in Japan

**DOI:** 10.3390/ijerph13080781

**Published:** 2016-08-03

**Authors:** Chorong Song, Harumi Ikei, Yoshifumi Miyazaki

**Affiliations:** Center for Environment, Health and Field Sciences, Chiba University, 6-2-1 Kashiwa-no-ha, Kashiwa, Chiba 277-0882, Japan; crsong1028@chiba-u.jp (C.S.); ikei0224@ffpri.affrc.go.jp (H.I.)

**Keywords:** natural environment, shinrin-yoku, forest bathing, urban green space, plant, wooden material, physiological relaxation, evidence-based medicine (EBM), preventive medicine, individual difference

## Abstract

Humans have evolved into what they are today after the passage of 6–7 million years. If we define the beginning of urbanization as the rise of the industrial revolution, less than 0.01% of our species’ history has been spent in modern surroundings. Humans have spent over 99.99% of their time living in the natural environment. The gap between the natural setting, for which our physiological functions are adapted, and the highly urbanized and artificial setting that we inhabit is a contributing cause of the “stress state” in modern people. In recent years, scientific evidence supporting the physiological effects of relaxation caused by natural stimuli has accumulated. This review aimed to objectively demonstrate the physiological effects of nature therapy. We have reviewed research in Japan related to the following: (1) the physiological effects of nature therapy, including those of forests, urban green space, plants, and wooden material and (2) the analyses of individual differences that arise therein. The search was conducted in the PubMed database using various keywords. We applied our inclusion/exclusion criteria and reviewed 52 articles. Scientific data assessing physiological indicators, such as brain activity, autonomic nervous activity, endocrine activity, and immune activity, are accumulating from field and laboratory experiments. We believe that nature therapy will play an increasingly important role in preventive medicine in the future.

## 1. Introduction

Despite living in this modern era and surroundings, our bodies are best adapted to living in a natural environment [1]. This could be because 6–7 million years ago our ancestors started evolving from a subset of primates into our current form [2], and early humans spent over 99.99% of that time living in a natural environment. If we define the beginning of urbanization as the rise of the industrial revolution, less than 0.01% of our species’ history has been spent in modern surroundings. The gap between natural settings, to which our physiological functions are best adapted, and the highly urbanized and artificial environment that we inhabit is a contributing cause of the “stress state” in modern people. In addition, the emergence of “megacities”, such as Tokyo and New York, which house more than 10 million inhabitants, has had an enormous influence on human lifestyles [3,4].

Rapid changes have also occurred in our environment over the last three decades, including the widespread use of computers. In 1984, American clinical psychologist Craig Brod coined the term “technostress” [5]. Other forms of technologies that expose us to more artificial elements have also contributed to the exacerbation of our stress levels.

As a result of these stressful situations, nature therapy, a health-promotion method that uses medically proven effects, such as relaxation by exposure to natural stimuli from forests, urban green spaces, plants, and natural wooden materials, is receiving increasing attention. It is empirically known that exposure to stimuli from natural sources induces a state of hyperawareness and hyperactivity of the parasympathetic nervous system that renders a person in a state of relaxation. This state becomes progressively recognized as the normal state that a person should be in and feel comfortable. Many conventional studies [6,7,8,9,10,11] have pointed out the benefits of nature therapy, but there is a lack of data that can shed light on these benefits on the body using evidence-based medicine (EBM), which is conventionally focused on physiological indications such as brain activity, autonomic nervous system activity, endocrine activity, and immune activity. Recently, the submission of scientific data on the basis of EBM was socially requested. In this review, we introduce the current situation on data accumulation.

**Figure 1:**
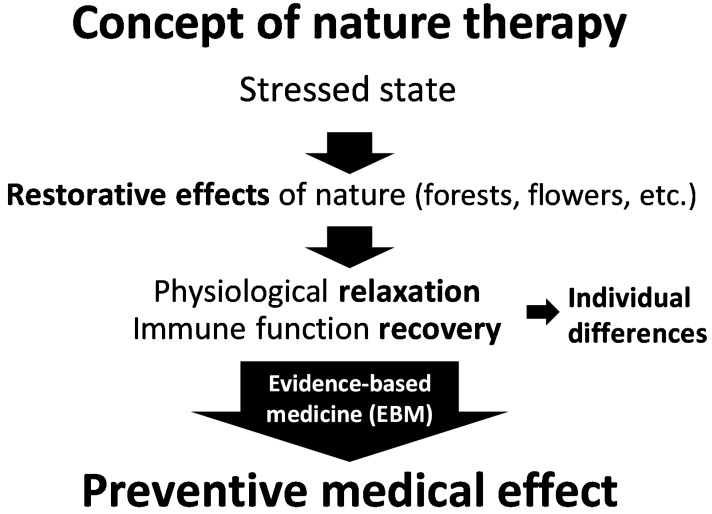
Concept of nature therapy [12].

Nature therapy is defined as “a set of practices aimed at achieving ‘preventive medical effects’ through exposure to natural stimuli that render a state of physiological relaxation and boost the weakened immune functions to prevent diseases” [12]. Unlike “specific effects” that are typically anticipated from pharmacological treatments, nature therapy seeks to improve immune functions, prevent illnesses, and maintain and promote health through exposure to nature, with the consequent attainment of a state of relaxation (Figure 1) [12,13].

In 1990, the first investigation on the physiological effects of being in a forest environment was performed in Japan [14,15,16], and active research has been conducted since. Therefore, this review aims to summarize research in Japan with regard to the following points: (1) activities in the central nervous system, autonomic nervous system, endocrine system, and immune system as functions of physiological relaxation as well as the immunity recovery effects of forest, urban green space, plants, and wooden material therapies along with (2) the analyses of individual differences that arise therein.

## 2. Methods

A scientific review of the accessible literature published over the last 20 years was conducted. Inclusion criteria for studies in this review were (1) studies conducted in Japan; (2) publication in the English language; and (3) publication from January 1996 to June 2016. Exclusion criteria for studies in this review were (1) review articles and (2) articles with only an abstract available. The search for relevant papers was conducted in the PubMed database with the keywords: “forest therapy”, “forest bathing”, “shinrin-yoku”, “urban park”, “urban green space”, “plant therapy”, “aroma therapy”, “horticultural therapy”, “foliage plants”, “fresh flowers”, “natural wooden material”, “Hinoki cypress”, “Japanese cypress”, “Japanese cedar”, and “physiological relaxation”. One thousand three-hundred and eight references were identified from this search. In addition, other publications cited in the collected papers were examined and added to the relevant literature. We applied our inclusion/exclusion criteria and retained 52 articles for our review.

Moreover, research related to the stimulation effects from nature in Japan includes forest, urban green space, plants (flowers and foliage plants), and wooden material effects, which we included in our study. The definition of each therapy (forest therapy, urban green space therapy, plant therapy, and wooden material therapy) indicates the therapeutic effect brought about by each of the stimulus.

## 3. Physiological Effects of Nature Therapy

### 3.1. Forest Therapy

In recent years, there has been considerable and increasing attention in using the forest environment as a place for recreation and health promotion. This approach is called “Shinrin-yoku” in Japan and means “taking in the forest atmosphere through all of our senses” [14]. It suggests “forest bathing”, which is a health promotion method that uses proven effects of a forest environment, such as relaxation, that can improve the health of the body and mind. In accordance with the accumulation of data, the idea of “forest therapy” has been proposed. Evidence-based “forest bathing (shinrin-yoku)” seeks preventive medical effects to improve weakened immune functions and prevent diseases by achieving a state of physiological relaxation through exposure to forest-origin stimuli.

In 1990, a preliminary study aimed to investigate the physiological effect of “Shinrin-yoku” using salivary cortisol levels, a marker of stress hormone [14,15,16]. Although the sample size was small, the result indicated that spending time in a forest environment can reduce stress state [16]. From 2005 to 2015, a physiological experiment was conducted over a one-week period on 744 participants in 62 forests located all over Japan. There are many reports related to this experiment describing indicators such as salivary cortisol levels [17,18,19,20,21,22,23,24], heart rate variability(HRV)-related sympathetic [22,23,24,25,26,27] and parasympathetic nervous activity [20,21,22,23,24,25,26,27], blood pressure [19,21,22,23,27], and pulse rate [19,20,21,22,23,24,27] to demonstrate the effects of relaxation.

In one particular study, the results on 280 participants (average age: 21.7 years) in 24 locations were reported [22]. The forest therapy experiment was conducted in forests representing the environmental characteristics of each region. The control experiment was conducted in an urban setting, such as stations and urban centers in large cities of the respective prefectures, following the same experimental schedule. The participants were 12 male university students who were residents of each region included in this experiment. The participants were divided into two groups of six persons each. On the first day, one group visited in the forest environment, whereas the other group visited in the urban environment; on the second day, the groups were switched. Upon arrival at the given site, the participants sat in chairs viewing the landscapes of their assigned areas. They also walked around their assigned areas. These activities were performed for about 15 min. Salivary cortisol levels, systolic and diastolic blood pressure, pulse rate, and HRV were measured. Saliva was collected utilizing a saliva collection tube, and cortisol was analyzed after storage in refrigerated and frozen environments (SRL Inc., Tokyo, Japan). Pulse rate and blood pressure were measured from the upper right arm using a digital sphygmomanometer based on the oscillometric method (HEM-1000, Omron, Tokyo, Japan). HRV was analyzed using the maximum entropy method (MemCalc/Win, GMS, Tokyo, Japan) after measuring the R-R interval (Activtracer AC-301A, GMS, Tokyo, Japan), setting the low-frequency component (LF) at 0.04–0.15 Hz and the high-frequency component (HF) at 0.15–0.40 Hz. The results indicated that the salivary cortisol level of participants was 13.4% lower after viewing the forest area compared to after viewing the urban area. The pulse rate and systolic and diastolic blood pressures were lower by 6.0%, 1.7%, and 1.6%, respectively. HF, which is known to reflect parasympathetic nervous activity, increased by 56.1% during forest therapy, and the LF/HF ratio, which is known to reflect sympathetic nervous activity, decreased by 18.0%. For the walking experiment, salivary cortisol levels were 15.8% lower after walking in the forest area compared to walking in the urban area. The pulse rate and systolic and diastolic blood pressures were lower by 3.9%, 1.9%, and 2.1%, respectively. HF increased by 102.0% and the LF/HF ratio decreased by 19.4%. The results after walking were very similar to those obtained in the seated position.

Based on these results, it can be concluded that forest therapy had the following effects: (1) it decreased the levels of salivary cortisol, a typical stress hormone; (2) it decreased the pulse rate; (3) it decreased the systolic and diastolic blood pressures; (4) it increased HF; and (5) it decreased the LF/HF ratio. These findings show that viewing or walking around a forest environment for a 15 min session of forest therapy induces a state of physiological relaxation. The results were almost identical to those of an experiment conducted on 420 participants in 35 locations [23].

In 2007, prefrontal cortex activity was measured using near-infrared time-resolved spectroscopy (TRS) [18]. Absolute hemoglobin concentrations in the prefrontal cortex were measured (TRS-l0; Hamamatsu Photonics K.K., Hamamatsu, Japan). The total hemoglobin concentration was lower after walking in a forest area than in a city area.

In contrast to most previous studies that experimented on healthy participants in their 20s, a study evaluated the effects of a 17 min forest walk on hypertensive middle-aged participants using HRV and heart rate as indicators [28]. Twenty hypertensive males (average age: 58.0 years; systolic blood pressure: 151.2 mmHg; diastolic blood pressure: 90.7 mmHg) participated. They walked in a coniferous forest that included many Japanese cypress trees (Akasawa natural recreation forest) and a corresponding urban area. The results showed that walking through the forest decreased heart rate and increased ln(HF) in comparison with walking in the urban area.

Next, nine hypertensive males (average age: 56.0 years) were studied to demonstrate the effects of a forest therapy program starting at 10:30 and ending at 15:05 [29]. Participants walked around their assigned area and then sat down and lay on their backs in the forest on waterproof sheets laid on the ground. This experiment was also conducted in a forest in Akasawa natural recreation forest, and physiological measurements were taken between 15:14 and 15:35. The measurements were taken at the same time on the day prior as a control to compare the experimental results with measures taken on a normal day. The comparison showed that forest therapy decreased the systolic blood pressure to 123.9 mmHg from the control value of 140.1 mmHg, and it decreased the diastolic blood pressure to 76.6 mmHg from the control value of 84.4 mmHg. Moreover, reductions in urine adrenaline and serum cortisol levels were also noted. These observations evidenced the physiological relaxation effects of a forest therapy program lasting a few hours on hypertensive male participants.

Furthermore, 17 middle-aged females (average age: 62.2 years) participated in a study with the same experimental design and locations the following year [30]. Similar results were obtained. Forest therapy elicited a decrease in pulse rate and salivary cortisol levels. There were substantial physiological benefits of forest therapy in middle-aged females.

Studies about patients with non-insulin-dependent diabetes also have been performed [31]. A total of 87 non-insulin-dependent diabetic patients (29 male and 58 female; average age: 61 years) walked in the forest nine times over a period of six years. Blood glucose levels of patients decreased after forest walking on all nine occasions. The mean blood glucose level showed a reduction from 179 mg × 100 mL^−1^ to 108 mg × 100 mL^−1^ after walking in the forest. This indicates that forest therapy has beneficial effects on blood glucose levels.

Three studies by Li et al. [32,33,34] demonstrated a boosting effect of forest therapy on weakened immune functions. Twelve male company employees aged between 37 and 55 years with weakened immune function were studied to observe the effects on natural killer (NK) cell activity. The forest therapy lasted two nights and three days [32]. The initial NK-cell activity value was measured at 8 a.m. upon arriving at the work environment three days before participation in the forest therapy. The participants traveled to the forest in the morning of Day 1 and took a walk in the forest for approximately 2 h in the afternoon, over a distance of 2.5 km. Blood samples were drawn at 8 a.m. on Day 2 as data for Day 1. The participants took a 2 h, 2.5 km walk in the morning and afternoon of Day 2. Again, blood samples were collected at 8 a.m. on Day 3 as data for Day 2. After the forest therapy, NK-cell activity had increased by 1.25 times on Day 1 and by 1.5 times on Day 2. Thus, this finding confirmed the immunity-boosting effects of forest therapy on NK cell activity. Another experiment with a similar design was conducted in the following year on male employees with weakened NK cell activity, and the results showed almost identical effects [33]. A similar two-night, three-day forest therapy experiment conducted on female nurses aged between 25 and 43 years also showed improvements in weakened NK cell activity, thereby demonstrating the similar effectiveness of this therapy in male and female participants [34].

Li et al. also studied the long-term effects of forest therapy [33,34]. Measures were taken at one week and one month after the male and female participants of the abovementioned experiments returned to work. High NK cell activity levels were maintained in both male and female groups at one week, and they were found to be maintained in the male group at one month.

To eliminate the effect of environmental change as a possible confounding factor, participants had a vacation of the same length of time to an urban area to test the effects of simple environmental change on NK cell activity [33]. The participants were males who underwent forest therapy, and the program involving the same hours and distance of walking as in the forest therapy group was replicated. The results of this experiment showed no improvement in weakened NK cell activity during the stay in the urban setting on Days 1 or 2.

From the above, the following effects of forest therapy were determined: (1) it improves weakened NK cell activity in male participants; (2) it shows the same effects in female participants; (3) these effects last in male group for a month; and (4) none of these improvements are observed in a population exposed to an urban setting.

In addition, several indoor studies focused on forest-derived olfactory stimuli exist. Hinoki cypress (*Chamaecyparis obtusa*), a coniferous tree, is a common and familiar tree in Japan. The effects of indoor exposure to Hinoki cypress wood oils on NK cell activity were examined [35]. The participants were 12 male instructors aged between 37 and 60 years who worked at a university. The experiment consisted of a three-night four-day stay in a metropolitan Tokyo hotel. The results showed that NK cell activity was increased and immune functions had improved. Therefore, olfactory stimulation brought about improvements in immune functions.

To investigate the effects of its leaf oil on brain activity and autonomic nervous activity, the experiment was conducted in an artificial climate chamber with the temperature, humidity, and illuminance set at 25 °C, 50%, and 230 lx, respectively [36]. A total of 13 female university students (average age: 21.5 years) were exposed to Hinoki cypress leaf oils, whereas a control group was not exposed to any odors (air). The odor was administered for 90 s, while participants sat with their eyes closed. Olfactory stimulation by Hinoki cypress leaf oil induced a reduction in oxyhemoglobin concentrations in the prefrontal cortex and increased parasympathetic nervous activity. Therefore, olfactory stimulation by Hinoki cypress leaf oil can induce physiological relaxation.

### 3.2. Urban Green Space Therapy

The effects of urban green space are now attracting attention as another source of nature in an accessible form. Recent demographic studies have found a positive association between exposure to urban green space and the perceived general health of residents [37]. Living in areas with accessible green spaces for walking also increases the longevity of senior citizens, independent of age, sex, marital status, baseline functional status, and socioeconomic status.

In a previous study [38], participants were asked to walk in an urban park during the spring season, and the control group was asked to walk in a street (hereinafter, urban setting) near the urban park. The temperature and humidity levels in the urban park were 24.7 °C and 39%, respectively, compared with 27.0 °C and 37% in the urban setting. The physiological indicators were HRV and heart rate, and the participants were 17 male university students (average age: 21.2 years). The participants were separated into groups of two for counterbalancing, and they walked in their respective environments for 15 min. The results showed that walking in the park increased ln(HF) and decreased the ln(LF/HF) ratio compared with walking in the urban setting. Decreased heart rates were also measured in the group that walked in the urban park. In conclusion, walking in an urban park in the spring (1) increases parasympathetic nervous activity; (2) inhibits sympathetic nervous activity; and (3) decreases the heart rate, thereby showing physiological relaxation effects.

The same experiment was performed in the fall (autumn) season [39]. The temperature and humidity levels in the urban park were 18.0 °C and 72%, respectively, compared with 19.2 °C and 65% in the urban setting. Twenty-three male university students (average age: 22.3 years) walked in an urban park and city area for 15 min. A brief walk in an urban park during fall induced parasympathetic nervous activity, suppressed sympathetic nervous activity, and decreased the heart rate.

The experiment was also conducted in the winter season [40] (temperature: 13.8 °C, humidity: 51%). Thirteen male university students (average age: 22.5 years), wearing protective gear against the cold with a hat, gloves, and other winter accessories, were asked to walk in the park. The results showed that the group that walked in the park showed an increase in ln(HF) and a decrease in the heart rate. From this, we can conclude that walking in a park has a physiological relaxation effect even in the winter.

Recently, urban gardening has also been attracting attention as another source of accessible exposure to nature. To study its effects, the physiological relaxation effects of viewing a kiwifruit garden were studied [41]. Seventeen adult female participants (average age: 46.1 years) went to a kiwifruit garden, sat on a chair, and appreciated the view for 10 min. The control group viewed buildings. The results revealed that the visual stimulus of the kiwifruit garden induced higher levels of ln(HF), increased parasympathetic nervous activity, and engendered a state of physiological relaxation.

Furthermore, elderly patients requiring long-term care were selected as participants to study the effects of seated viewing of the forest, which was constructed on a hospital rooftop. This experiment showed increased parasympathetic nervous activity and decreased sympathetic nervous activity compared with the parking lot (control) on the first floor [42].

### 3.3. Plant Therapy

Through experience, we are all aware of the physiological relaxing effects of exposure to flowers, such as roses and foliage plants that are frequently used in flower arrangement. However, according to EBM, there is no scientific data based on physiological indicators to support the various physiological effects of plant therapy. Here we present data on the prefrontal cortex activity and autonomic nervous activity that were obtained in response to two categories of stimuli: visual and olfactory stimuli.

#### 3.3.1. Visual Stimulation Experiment

Here, we explore the physiological relaxation effects of exposure to a visual stimulus of fresh roses. One study included a total of 114 participants, including 55 high-school students (37 males and 18 females, average age: 15.5 years), 14 medical workers (14 females, average age: 42.1 years), and 45 office workers (31 males and 14 females, average age: 38.0 years). The stimulus consisted of 30 pink, odorless roses (*Rosa*, cultivar name: Dekora), 40 cm in length. The distance between the roses and the participants was set at approximately 37–40 cm, and the duration of exposure to the visual stimulus was 4 min. The physiological indicators employed as the study parameters were HRV, measured by fingertip accelerated plethysmography (ARTETT, U-Medica Inc., Osaka, Japan), and pulse rate, calculated by a 60/a-a interval. HF increased by 16.7% and the LF/HF ratio decreased by 30.5% in the 55 high school students in response to the visual stimulus of fresh flowers. HF also increased by 33.1% in the 14 medical workers and by 21.4% in the male office worker group [43]. The results on the total group of 114 participants showed an average increase of 15.1% in HF and a decrease of 16.3% in the LF/HF ratio. From this, we can conclude that visual stimulation with odorless fresh roses (1) increases parasympathetic nervous activity to render a state of relaxation and (2) decreases sympathetic nervous activity to alleviate stress state.

Furthermore, the relaxation effects of foliage plants commonly found in homes and offices were studied in a total of 85 high-school students (41 males and 44 females, average age: 16.5 years) [44]. The houseplants used as the visual stimuli consisted of three pots of the striped dracaena (*Dracaena deremensis*), which stood at a height of approximately 55–60 cm from the bottom of the pot. The participants were exposed to the visual stimulus of the plants for 3 min at a distance of approximately 55 cm from the plants. The results showed that HF of the participants increased by 13.5% compared with that of the controls not exposed to the plants. The LF/(LF + HF) ratio, which reflects sympathetic nervous activity, decreased by 5.6% compared with that of the controls. Again, visual stimulation with dracaena, a common foliage plant, was proven to have physiological relaxation effects.

Next, an experiment was conducted to compare the effects of fresh and artificial pansies [45]. The participants were 40 high-school students (19 males and 21 females, average age: 16.4 years). Fresh and artificial yellow pansies grown or mounted in a planter were employed as the visual stimuli for 3 min. Compared with artificial pansies, visual stimulation with fresh pansies resulted in a decrease in the LF/HF ratio, thus demonstrating the alleviation of stress state.

Subsequently, the physiological relaxation effects of three-dimensional (3D) images of nature were studied [46]. Visual stimulation by exposure to images depicting nature is a convenient means to get in contact with nature in our modern, stress-laden society. As such, realistic 3D images are becoming popular. Visual stimulation with 3D images of natural sceneries was expected to have a different effect on the body compared with stimulation by two-dimensional (2D) images. Images of water lilies in 2D and 3D were shown for 90 s each to 19 male participants (average age: 22.2 years). The visual stimulus measured 1.7 × 3.0 (H × W) m, and the distance between the display and the participants was set at 2.5 m. Autonomic nervous activity by HRV and prefrontal cortex activity by near-infrared spectroscopy were measured as indicators of the physiological response. In HRV, the R-R interval was measured on a portable electrocardiographic monitor, and the maximum entropy method (MemCalc/Win, GMS, Tokyo, Japan) was used for analysis. The right and left prefrontal cortices were measured for oxygenated hemoglobin levels using near-infrared spectroscopy (NIRO-200, Hamamatsu Photonics K.K., Hamamatsu, Japan). The results showed that exposure to realistic 3D images of nature decreased ln(LF/HF) and decreased oxygenated hemoglobin levels in the right prefrontal cortex compared with 2D images, thereby showing the physiological relaxation effects of this form of natural visual stimulation.

We also compared the activation of the prefrontal cortex during presentation of real foliage plants with a projected image of the same foliage plants [47]. While viewing the plants and images, oxy-hemoglobin (oxy-Hb) concentrations in the prefrontal cortex were measured using near-infrared time-resolved spectroscopy measured by the TRS-20 system (Hamamatsu Photonics K.K., Hamamatsu, Japan). Compared with a projected image of foliage plants, viewing the actual foliage plants increased oxy-hemoglobin concentrations in the prefrontal cortex. Humans responded differently to the presentation of actual plants compared with images of these plants.

#### 3.3.2. Olfactory Stimulation Experiment

The physiological effects of olfactory stimulation with rose and orange essential oils were studied [48] in 20 female university students (average age: 22.5 years). The stimulus was the scent of the essential oils made from roses and orange zest. The stimulation was counterbalanced with no odor (air) as the control. The concentration of the essential oils was adjusted to vary from “slight smell” to “weak smell”, and the duration of the stimulation was 90 s. The physiological indicator was the level of oxygenated hemoglobin in the prefrontal cortex, measured using near-infrared time-resolved spectroscopy (Hamamatsu Photonics K.K., TRS-20, Hamamatsu, Japan). The results showed that olfactory stimulation by essential oils from roses and orange zest decreased oxygenated hemoglobin levels in the right prefrontal cortex.

Next, the relaxation effects of olfactory stimulation with fresh roses were studied [49]. The strength of perceptibility of the stimulus was determined as “weak” or “easily” sensed and the duration of stimulation was 90 s. Increased HF and a trend toward a decrease in LF/HF were observed over the 90 s interval as a result of olfactory stimulation with fresh roses.

Furthermore, the effects of olfactory stimulation with essential oils of perilla were also studied [50]. Perilla is an herb that has been used as an accompaniment to sashimi and other dishes in Asia and has also been widely employed for its medicinal value. It is a common ingredient in antidepressant medications in traditional Chinese medicine and is thus used as a medicinal herb in modern times as well. However, much of its physiological relaxation effects are yet to be defined. To research the effects of this olfactory stimulus, 19 female university students (average age: 21.6 years) were exposed to perilla essential oils, whereas the control group was not exposed to any odors (air). The concentration of the essential oil was determined as “weak” or “easily” sensed and the duration of the stimulation was 90 s. The physiological indicator was the concentration of oxygenated hemoglobin in the prefrontal cortex, measured using near-infrared time-resolved spectroscopy (Hamamatsu Photonics K.K., TRS-20, Hamamatsu, Japan). The results showed that oxygenated hemoglobin levels in both the right and left prefrontal cortices decreased in the last third of the 90 s period in the participants compared with the control group. Thus, these results demonstrate the calming effects of olfactory stimulation with perilla essential oils on prefrontal cortex activity.

### 3.4. Wooden Material Therapy

Wood is a natural material that has been deeply rooted in Japanese sensibilities. Empirically, it has been perceived as a material with relaxing properties. However, the accumulation of data on the activities of the brain, autonomic nervous system, and endocrine and immune systems supported by EBM principles is extremely limited.

Here, physiological responses to stimulation with wood will be shown by presenting experiments that were primarily conducted indoors.

#### 3.4.1. Visual Stimulation Experiment

Previous studies have reported on the physiological changes caused by visual stimulation with wooden materials in indoor rooms and its effect on continuous blood pressure and pulse rate as physiological indicators [51,52,53]. An actual room (13 m^2^) was built for the study, and the effects of visual stimulation were measured over a 90 s stimulation period as the percentage of wood in the structure and design of the room changed. Common wooden living rooms in Japan contain approximately 30% wood in their structure. A common wooden living room (30% wood) and a living room with extra wood added to the walls (45% wood) were compared. Reductions in pulse rate and diastolic blood pressure were observed, indicating that common wooden living rooms containing 30% wood have visually calming physiological effects. An increase in pulse rate was observed in living rooms containing 45% wood. In a 30% wooden room with the addition of wooden pillars and crossbeams, the pulse rate increased in a manner similar to that in the room with 45% wood, thereby showing a state of physiological wakefulness. From the above, it was clarified that the physiological changes were induced by variation in the wood percentage or design in a wooden living room.

Sakuragawa et al. [54] have demonstrated the different physiological responses to visual stimulation of full-sized Hinoki wall panels and a white steel wall panel (control). Fourteen male college students viewed both wall panels for 90 s. Participants were also asked to rate the wall panels according to whether they liked them or not. During the visual stimulation from the Hinoki wall panels, systolic blood pressure decreased in the participant group that evaluated the Hinoki wall panels as “like“, and there was no change in the participant group that evaluated them as “dislike“. For the white steel wall panels, systolic blood pressure increased in the participant group that evaluated them as “dislike”.

#### 3.4.2. Olfactory Stimulation Experiment

Japanese cedar and Japanese cypress wood chips, essential oil, and essential oil constituents were used as substances for olfactory stimulation. The odors were administered by means of an essential oil inhalation device. The experiment was conducted on participants seated in an indoor artificial climate chamber with the temperature, humidity, and illuminance set at 25 °C, 60%, and 50 lx, respectively. The strength of perceptibility of the stimulus was adjusted to a “weak smell”, and the duration of the stimulation was approximately 60–90 s. The results showed a decreased systolic blood pressure and a calming effect on the prefrontal cortex activity in response to olfactory stimulation with cedar materials and cypress chips; therefore, this olfactory stimulation had an overall physiologically relaxing effect [55].

It is reported that volatile organic compounds (VOCs) emitted from Japanese cedar (*Cryptomeria japonica*) interior walls induce physiological relaxation [56]. Sixteen male university students (average age: 23.5 years) performed arithmetic tasks both in the absence (control) and the presence of VOCs emitted from the Japanese cedar for repeated cycles of 15 min of work and 5 min of rest. Salivary chromogranin A (CgA) concentration at the post-work measurement was higher compared with the pre-work measurement under the control condition. However, the change under the Japanese cedar condition between pre- and post-work measurements was not significant. The authors are suggesting that VOCs emitted from Japanese cedar suppresses activation of an increase in salivary CgA secretion. Furthermore, the effects of the essential oil of the Pinaceae (*Abies sibirica*) during and after performance of a sustained task on a visual display terminal (VDT) have been examined [57]. Nine male university students (average age: 22 years) performed the VDT work for 30 min twice, at first in the absence (control) and next in the presence of volatiles from the essential oil of Pinaceae. After VDT work, the mean R-R intervals were higher in the Pinaceae condition than the control. This meant that the heart rate was slower. There were significant differences in electroencephalogram (EEG) results as well. The EEGs recorded from the C3 and C4 electrode sites of the international 10–20 system were analyzed. After VDT work, the theta band powers were increased and the alpha band powers were decreased in the Pinaceae condition compared with the control. The arousal level from VDT work was decreased by the essential oil of Pinaceae.

On the other hand, differential results have also been reported. The influence of olfactory stimulation of Hiba (*Thujopsis dolabrata*) odor on contingent negative variation (CNV) was investigated [58]. Five milliliters of undiluted Hiba oil were warmed in an oil warmer with a 15 W incandescent lamp. In the control condition, only the lamp was on. Sixteen females were exposed to the Hiba oil and control conditions for over 30 min. Compared to the control, the amplitude of the CNV components were larger and reaction time to the imperative stimulus was shorter in the Hiba oil condition. Although the sensory intensity was not indicated, there is a possibility that the inhalation concentration of the Hiba oil was high.

Single substance inhalation experiments of α-pinene and limonene, which are the main volatile components of most wood materials and forests, have also been conducted. The blood pressure of the participants was measured every second throughout the 90 s duration of stimulation. The results showed that inhalation of α-pinene and limonene decreased systolic blood pressure [55]. Furthermore, increased parasympathetic nervous activity and decreased heart rate were also reported on olfactory stimulation with limonene [59].

As a natural construction material, wood normally needs to be thoroughly dried before use in order to prevent warping, and this is most often done by artificial drying methods. However, these processes degrade the “natural scent of wood” by altering the natural properties of the wood constituents by exposure to high temperatures or by loss of the low boiling point components. Therefore, the effects of inhaling odorous substances from chips made of “air-dried cypress wood” (produced through natural drying processes to preserve the “scent of wood”) and “high-temperature-dried cypress wood” (exposed to high-temperature, high-speed drying) were compared and shed light on the differences in the effects on the left and right prefrontal cortex activity [60]. The experiment was conducted with 19 female university students (average age: 22.5 years) in an artificial climatic chamber. The strengths of perceptibility of the stimuli were adjusted to vary from “slight smell” to “weak smell”. The results demonstrated that olfactory stimulation with “air-dried cypress wood” had a greater calming effect on the left and right prefrontal cortices of participants compared with the inhalation of odorous substances originating from “high-temperature-dried cypress wood”.

#### 3.4.3. Tactile Stimulation Experiment

A study on the effects of contact with wood or artificial substances on the systolic blood pressure [61] has previously been conducted. It was shown that contact with a metal board caused great fluctuations in the systolic blood pressure, whereas contact with cypress and cedar wood caused little fluctuation.

Sakuragawa et al. [62] have demonstrated the following: (1) contact with a metal board increased blood pressure, but the increase was inhibited when the metal was warmed; (2) contact with an acrylic board increased blood pressure and the rate of increase in blood pressure was greater when the acrylic board was chilled; and (3) blood pressure did not change in response to contact with cedar, cypress, or oak materials. Furthermore, it did not increase even if the oak material was chilled. These results showed that temperature conditions have a sizable effect on blood pressure increases if contact is made with artificial materials, such as metals and acrylic, whereas contact with wood does not cause physiological stress, which manifests itself in the form of blood pressure increase, regardless of its temperature. This was true whether tested at room or lower temperature, thereby showing the superiority of nature-derived wood as a material.

## 4. Individual Differences in the Physiological Relaxation Effects of Nature Therapy

It is known that great individual differences are observed in physiological data collected in research on stress and relaxation, but few methods have been proposed to elucidate this variability.

We used two methods to elucidate the variability phenomenon: (1) “law of initial value” and (2) “personalities”, such as “Type-A behavioral patterns” and “trait anxiety”. We introduce the approach method and analyzed data below.

### 4.1. Approach of the “Law of Initial Value”

The “law of initial value” was advocated by Wilder [63,64], and it describes the principle that the direction of the response to a stimulus depends largely on the initial value. Therefore, the higher the initial value, the smaller the response to function-raising stimuli and the larger the response to function-depressing stimuli. Lacey [65] examined the correlation between initial values and changes in systolic/diastolic blood pressure and heart rate caused by a stressor and reported that participants whose initial values were high responded poorly to function-raising stimuli, such as a stressor. Hord et al. [66] demonstrated the relationship between initial values and changes in heart rate and respiratory rate caused by a stressor. They found that there were significant correlations between initial values and changes in heart rate and respiratory rate. Previous studies have been conducted mainly with regard to the relationship to the response by function-raising stimuli and the initial value [65,66,67,68,69]. Studies to clarify the relationship between the initial value and the response by function-depressing stimuli, such as natural therapy, hardly exist.

This research investigated the individual differences in physiological relaxation effects from forest therapy from the perspective of “law of initial value” with the purpose of clarifying the physiological adjustment effects of forest therapy [70]. The experiment was conducted between 2012 and 2013 in forest and urban areas in eight locations across Japan. The indicators measured were blood pressure and pulse rate. The sample in each experiment location included 12 male university students in their 20s, and a total of 92 participants for whom data could be obtained (average age: 21.5 years). They were randomly assigned to two groups of six persons each. One group spent Day 1 in the forest and the other in the urban area; on Day 2, the two groups switched the experiment locations. Commercial areas around Japan Railway stations were selected as the urban areas, and participants took 15 min walks in the forest and urban areas.

**Figure 2:**
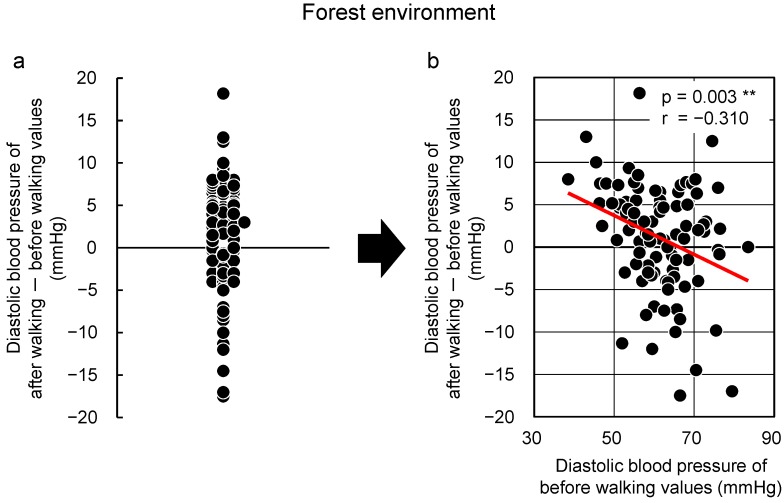
Changes observed with respect to walking in a forested area. Individual differences (**a**) and the relationship between the “initial value” and the “changes after walking in a forested area” (**b**) with respect to diastolic blood pressure (*n* = 92). ** *p* < 0.01 by Pearson correlation test [70].

**Figure 3:**
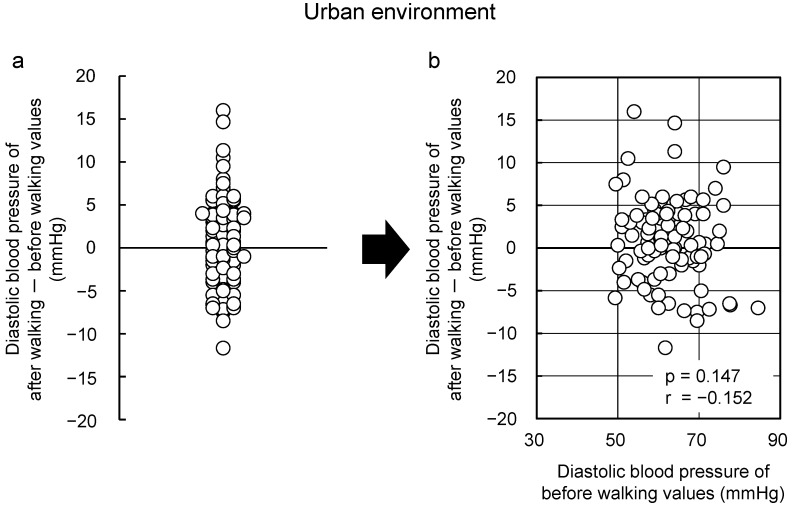
Changes observed with respect to walking in an urban area. Individual differences (**a**) and the relationships between the “initial value” and the “changes after walking in an urban area” (**b**) with respect to diastolic blood pressure (*n* = 92). Pearson correlation test [70].

More participants presented with blood pressure and pulse rate reductions from walking in the forest; however, these parameters also increased in some participants, thereby showing that there is great individual variation (Figure 2a). First, the diastolic blood pressure was studied on the basis of the “law of initial value”. Figure 2b shows the relationship between “initial value (absolute value before walking in the forest)” and “the change (value after walking in the forest)–(value before walking in the forest)”. As shown in this figure, there is a negative correlation between the “initial value” and the “change”, showing that values decreased by walking in the forest in participants whose initial values were high, and values increased in participants with low initial values. Meanwhile, there was no correlation between the “initial value” and the “change” in Figure 3, which shows the results of the same participants in the urban area. The results were similar in walking the relationship between the “initial value” and the “change” for pulse rate. In other words, it was concluded that walking in the forest has physiological adjustment effects that bring the diastolic blood pressure and pulse rate closer to the ideal values.

Conversely, in terms of previous studies on the “law of initial value”, Tsunetsugu and Miyazaki [71] measured salivary cortisol levels before and after a 15 min walk in the forest and reported a negative correlation between the initial value and the change. Lee et al. [13] also showed a negative correlation between the values before and after a 15 min walk in the forest and changes in salivary immunoglobulin A (IgA) levels. IgA levels decreased in the group with a higher initial value, and there was a negative correlation between the initial value and the change after forest therapy.

### 4.2. Approach Employing “Personality”

Past studies on individual variations have approached the topic through a behavioral pattern category called the “Type-A behavior pattern” [72,73,74]. The Type-A behavior pattern can be defined as an overt behavioral syndrome related to a lifestyle that is characterized by excessive competitiveness, striving for achievement, aggressiveness, time urgency, acceleration of common activities, restlessness, hostility, hyperalertness, explosiveness of speech amplitude, facial musculature tension, feelings of struggling against the limitations of time, and insensitivity to the environment [75,76].

Here, “personality” was used to organize differences in individual physiological responses to forest therapy. In seated forest-viewing experiments conducted in 44 locations nationwide, 485 participants were divided into Type-A and Type-B groups. The differences in their responses were studied [73]. The results showed that 15 min of seated viewing of the forest decreased the pulse rate by 2.2 beats/min in the 485 participants. Compared with the reduction rate of 1.6 beats/min in the urban areas, the forest group showed a remarkable 0.6 beat/min difference in the reduction of pulse rate. Next, the participants were categorized into two groups: Type-A and Type-B behavior patterns (233 and 252 participants, respectively). The pulse rate of the Type-B group decreased from 65.7 beats/min to 63.2 beats/min, showing a 2.5 beats/min decrease from seated viewing of the forest, whereas no significant change was observed in the Type-A group. Finally, the Type-A and Type-B groups were divided again to form four subgroups. The low-score Type-B group showed a decrease of 1.7 beats/min in the forest compared with that in the urban area; however, in the other three groups, there was no significant change between the forest and urban area groups. In conclusion, in comparison with the urban area, the change in the pulse rate from 15 min of seated viewing in the forest (1) greatly decreased the heart rate in all 485 participants; (2) it largely decreased the heart rate in Type-B participants, but no difference was observed in the Type-A participants; and (3) after dividing the whole group into four subgroups, a large reduction was observed only in the low-score Type-B group. On the basis of these results, the study demonstrated that forest therapy can decrease the pulse rate but this reduction depends largely on the participant’s personality; furthermore, individual variability related to changes in pulse rate in the forest therapy may be explained by personality categories.

## 5. Conclusions

This review presented scientific data to elucidate the physiological relaxation effects of nature therapy on activities of the central nervous system, autonomic nervous system, endocrine system, and immune system on the basis of advances in various physiological indicators from the viewpoint of EBM in Japan. Furthermore, our methods to approaching individual differences that arise in these measures were also described.

In Japan, the therapeutic effects of the stimulation of nature have always been known empirically, but because of the lack of data, submission of scientific data was socially demanded, and since then, there has been a gradual progression. The present review showed the current state of data accumulation. However, several limitations exist. First, most of the studies use short time periods of stimulation. In the future, long-term data over days, weeks, and months are needed. Second, there are many studies on men and women in their 20 s. To generalize the findings, further studies based on a larger sample, including various age groups, are required. In addition, studies in people with premorbid conditions are also required. Finally, it is necessary to comprehensively evaluate the parameters that are used as indicators of brain activity, autonomic nervous activity, endocrine activity, and immunization activity.

Considering the significance of quality of life in our modern stressful society, the importance of nature therapy will further increase. The therapeutic effects of natural stimulation suggest a simple, accessible, and cost-effective method to improve the quality of life and health of modern people. Moreover, the elucidation of these physiological effects from the viewpoint of EBM is an important task for the future.

## Abbreviations

The following abbreviations are used in this manuscript:

2D	Two-dimensional
3D	Three-dimensional
EBM	Evidence-based medicine
EEG	Electroencephalogram
HRV	Heart rate variability
IgA	Immunoglobulin A
NK	Natural killer
LF	Low frequency
HF	High frequency
SD	Semantic differential
TRS	Near-infrared time-resolved spectroscopy
VOCs	Volatile organic compounds
VDT	Visual display terminal
